# Production and Characterization of Cellulosic Pulp from Mango Agro-Industrial Waste and Potential Applications

**DOI:** 10.3390/polym15153163

**Published:** 2023-07-26

**Authors:** Maribel García-Mahecha, Herlinda Soto-Valdez, Elizabeth Peralta, Elizabeth Carvajal-Millan, Tomás Jesús Madera-Santana, María Guadalupe Lomelí-Ramírez, Citlali Colín-Chávez

**Affiliations:** 1 Coordinación de Tecnología de Alimentos de Origen Vegetal, Centro de Investigación en Alimentación y Desarrollo, A.C. (CIAD), Carretera Gustavo Enrique Astiazarán Rosas, No. 46, Col. La Victoria, Hermosillo 83304, Sonora, Mexico; maribel.garcia.dc19@estudiantes.ciad.mx (M.G.-M.); eperalta@ciad.mx (E.P.); madera@ciad.mx (T.J.M.-S.); 2 Coordinación de Tecnología de Alimentos de Origen Animal, Centro de Investigación para Alimentación y Desarrollo, A.C. (CIAD), Carretera Gustavo Enrique Astiazarán Rosas, No. 46, Col. La Victoria, Hermosillo 83304, Sonora, Mexico; ecarvajal@ciad.mx; 3 Departamento de Madera, Celulosa y Papel del Centro Universitario de Ciencias Exactas e Ingenierías, Universidad de Guadalajara, Km 15.5 Carretera Guadalajara-Nogales, Zapopan 45220, Jalisco, Mexico; maria.lramirez@academicos.udg.mx; 4 Centro de Innovación y Desarrollo Agroalimentario de Michoacán, A.C. (CIDAM), Km 8 Antigua Carretera a Pátzcuaro s/n, Morelia 58341, Michoacán, Mexico; citllalina2982@gmail.com

**Keywords:** cellulosic pulp, mango waste, tegument fibers, alkali method, acid method

## Abstract

The growing demand for cellulosic pulp presents an opportunity to explore alternatives to this material, focusing on utilizing agro-industrial residues. Mango’s tegument is a rich source of cellulose, making it a valuable raw material for manufacturing single-use articles or blends with biopolymers. In this sense, employing conventional alkaline and acid chemical treatments, the mango’s tegument was treated to obtain cellulosic pulp. The teguments were subjected to treatment with alkaline solutions (2% and 4% NaOH *w*/*v*) at 80 °C for 1 or 2 h or with an acetic acid solution (1:1 or 1:2 CH_3_COOH:H_2_O_2_) at 60–70 °C for 1 or 2 h. After treatment, an evaluation was conducted to assess the yield, color, chemical analysis, and structural, thermal, and morphological properties. The alkali treatments produced cellulosic pulps with a light color with 37–42% yield and reduced hemicellulose content. The acid treatments produced orange–brown cellulosic pulp with 47–48% yield and higher hemicellulose content. The acid pulps were thermally more stable (maximum decomposition at 348–357 °C) than the alkali pulps (maximum decomposition at 316–321 °C). The crystallinity index demonstrated that both treatments increased the crystallinity of the cellulose pulps compared with the untreated tegument. The thermal stability of cellulosic pulp at the processing temperatures of disposable tableware (50–120 °C) revealed that plates, bowls, trays, and cups could be produced. Another potential application is as a component of blends with biopolymers to make straws or rigid food packaging (trays) with reinforced structures.

## 1. Introduction

Takeaway consumer items comprise the largest environmental waste share (50% to 88%), mainly consisting in plastic [1], and the COVID-19 pandemic has led to a sharp rise in the demand for takeaway food services. Consequently, environmental policies and consumer preferences promote an increased need for cellulosic pulp to produce biodegradable single-use items such as disposable plates, bowls, and cups. The expansion of e-commerce has increased the demand for cardboard and corrugated packaging, also made of cellulosic pulp [2]. By 2040, cellulosic pulp demand is expected to increase significantly due to its better biodegradability and recyclability than plastics [3,4]. Cellulosic pulp is traditionally obtained from wood and crops through physical, chemical, and physicochemical treatments. Acid and alkali treatments are widely employed as chemical methods to obtain cellulosic pulp, and a minimal environmental impact can be obtained by implementing integrated physicochemical and biological post-treatments on wastewater. Thereby, a combination of two or more processes such as sedimentation, floatation, filtration, adsorption, reverse osmosis, wet oxidation, and biological treatments (fungi, bacteria, algae, and enzymes) can be used for removing suspended matters and toxic components [5]. The treatments modify the fiber’s surface and morphology, and remove lignin and hemicellulose to improve the physicochemical properties of cellulosic pulp. The treatments are different in regard to their action mechanisms. Alkali treatment breaks the ester bond between hemicellulose and lignin and remove a portion of lignin, a part of hemicelluloses, wax, and oils. Acid treatment facilitates the hydrolysis of glycosidic bonds between hemicelluloses and cellulose, breaking cellulose and hemicellulose chains into individual sugar monomers. Additionally, this process effectively removes organic or aqueous extractives without significantly affecting the lignin content [6,7].

Agro-industrial waste is another possible source of cellulosic pulp. Apple pomace, pineapple, and orange peels have been reported as raw materials that can be used to produce disposable cups by the soda-pulping process. The application depends on the chemical composition, linkages, and inter and intramolecular bonds [8,9]. Mango (*Mangifera indica* L.) is the most predominant tropical fruit in the world, with a production of 41 million tons in 2020 [10]. Depending on the mango variety, 33–85% is the flesh, 7–24% peel, and 9–40% seed (*w*/*w*), with peel and seed being the waste for valorization [11,12]. Mango processing generates 35–60% (*w*/*w*) agro-industrial waste, and its inappropriate disposal causes environmental contamination. This waste comprises carbohydrates such as cellulose, hemicellulose, lignin, starch, pectin, and other minor components [9,13]. Mango’s oblong seed has a fibrous surface called the tegument covering the embryo (kernel). The tegument comprises mainly fibers (cellulose, hemicellulose, and lignin) with the potential of being converted into cellulosic pulp, the raw material used to produce molded single-use trays, plates, cups, and bowls for the food service industry. Therefore, mango’s tegument from mango-industry wastes could be a novel source of raw materials to produce cellulosic pulp with low environmental impact and contribute to the valorization of this agro-industrial waste. This work presents the use of mango’s tegument in the production of cellulosic pulp. Two different methods were used (alkali and acid) to study their effects on cellulosic pulp’s yield and chemical, physical, and morphological properties.

## 2. Materials and Methods

### 2.1. Materials

Mango waste (var. Tommy Atkins) was kindly provided by Fruxo, an agro-industry located in Tepic, Nayarit, Mexico. The waste batch was frozen at −20 °C until use. Then, the mango’s tegument was separated from the kernel, and the samples were cut into 2–4 cm^2^ and freeze-dried. The freeze-dried tegument was divided into three batches: one was subjected to alkali treatments, the second to acid treatments, and the third was the raw material not treated (untreated fibers or control). Sodium hydroxide (NaOH), hydrogen peroxide (H_2_O_2_), sulfuric acid (H_2_SO_4_), and acetone (C_3_H_6_O) were purchased from Fagalab^®^, Mocorito, Sinaloa, Mexico; acetic acid (CH_3_COOH) J.T. Baker was from CTR Scientific, Monterrey, N.L., Mexico; and sodium chlorite (NaClO_2_) Golden Bell was from Proquisur, CDMX, Ciudad de México, Mexico.

### 2.2. Proximate Analysis of Mango’s Tegument

The tegument was characterized following the protocols of the AOAC [14] in terms of moisture content (934.01), fat (920.39), protein (960.52), ash (942.05), and total dietary fiber (985.29). The analysis was performed in triplicate, and the carbohydrate content was calculated by the difference in Equation (1):

(1) $$\%\;carbohydrates=100-\left(\%\;ash+\%\;fat+\%\;protein\right)$$

### 2.3. Cellulosic Pulp: Alkali Treatment

The production of cellulosic pulp from the first batch of tegument was treated according to Cordeiro et al. (2014) [15], with some modifications. Pieces of tegument were ground using a food processor and added to alkaline solutions (2% and 4% NaOH *w*/*v*) at a 1:20 (*p*/*v*) ratio (fiber:solution) at 80 °C for 60 or 120 min with constant agitation. Subsequently, the fibers were washed with distilled water by vacuum filtration until a pH of seven was obtained, and then they were dried at 50 °C for 24 h. Then, alkali-treated fibers were bleached for 2 h at 50 °C with a 1:20 solution (fiber:solution) composed of 30% H_2_O_2_ (*v*/*v*) and 4% NaOH (*w*/*v*). The fibers were washed with distilled water to a neutral pH and dried at 50 °C for 24 h. The bleached fibers were packed in sealed plastic bags and stored at room temperature under controlled humidity (RH 26%). Four treatments were performed, and their descriptions are shown in Table 1.

### 2.4. Cellulosic Pulp: Acid Treatment

The cellulosic pulp produced by the acid treatment was processed according to Curtis Patiño (1986) and Kiaei et al. (2014) [16,17]. Pieces of tegument were ground using a food processor, and they were added to the acetic acid solution (1:1 or 1:2 CH_3_COOH:H_2_O_2_) at a 1:20 (*p*/*v*) ratio (fiber:solution) at 60 or 70 °C for 1 or 2 h with constant agitation. Subsequently, the fibers were washed with distilled water, filtered under vacuum to a neutral pH, and dried at 50 °C for 24 h. The acid fibers were packed and stored at room temperature under controlled humidity (RH 26%). Eight acid treatments were performed (Table 1).

### 2.5. Yield of the Pulping Process

The cellulosic pulp yield (% *w*/*w*) was calculated according to Equation (2):

(2) $$Yield=\frac{W_{f}}{W_{i}}\times 100\%$$

where *W_f_* and *W_i_* are the final dry weight of cellulosic-treated fibers and the initial dry weight of untreated freeze-dried tegument, respectively [18].

### 2.6. Color Analysis

The effect of the treatments on the cellulosic pulp was evaluated using the color measured with the CIELAB system with a colorimeter (Konica Minolta CR 400, Ramsey, NJ, USA). In this system, coordinate L* represents lightness (0 black and 100 white), coordinate a* represents redness (−a green and +a red), and coordinate b* represents yellowness (−b yellow and +b blue). The Hue (H) value is used to define the difference between a specific color with reference to a gray color with the same lightness. Three replicates were analyzed for each treatment, and the results are expressed as the average ± standard deviation [19].

### 2.7. Chemical Analysis of the Fibers

To evaluate the holocellulose content, the samples were first subjected to moisture removal. After that, extractives in organic and aqueous solvents were removed to avoid interference in the holocellulose determination according to Tappi Test Methods T 260 om-07 [20]. The cellulosic pulps were extracted with acetone in a Soxhlet extractor for two h after the first siphon. Subsequently, the cellulosic pulp was extracted with boiling water for sixty minutes to separate tannins, sugars, and gums from the fibers to obtain the organic extractive-free pulp. According to ASTM D-1104, the holocellulose determination was carried out with the extractive-free samples treated with sodium chlorite and glacial acetic acid at 70 °C [21,22].

Lignin content was determined according to Tappi Test Methods T 222 om-21 [23]. Selected samples were subjected to a two-step acid hydrolysis. First, the extractive-free samples were treated with concentrated sulfuric acid (72%), and then the samples were treated with diluted sulfuric acid (4%) [22].

### 2.8. Fourier Transform Infrared Spectroscopy (FTIR) of the Fibers

FTIR spectra of the cellulosic pulps were analyzed using a Thermo Fisher Scientific Model Nicolet iS50 FT-IR (Madison, WI, USA). Scans were collected from 4000 to 400 cm^−1^ to obtain the spectra (transmittance mode, 32 scans, and a resolution of 4 cm^−1^). Samples of cellulosic pulp were mixed with dry KBr (1:20 *w*/*w*) to prepare tablets using a hydraulic press [15,24]. See Figure S1 (Supplementary Material).

### 2.9. Thermogravimetric Analysis (TGA) of the Fibers

The decomposition of cellulosic pulp by the effect of temperature was evaluated by thermogravimetric analysis using a TA Instruments thermogravimetric balance model TGA-Discovery (New Castle, DE, USA). The samples were placed in alumina crucibles and heated from 25 to 600 °C at a heating rate of 10 °C/min under a nitrogen atmosphere at a 25 mL/min flow rate. Each treatment’s mass loss and differential thermogravimetric (DTG) thermograms were registered.

### 2.10. X-ray Diffraction Analysis (XRD) of the Fibers

The XRD analysis was performed by X-ray powder diffraction (XRD, D2 Phaser (Bruker, Karlsruhe, Germany)) with radiation CuKα (Kα1 = 1.5406 Å and Kα2 = 1.5444 Å, and the relation Kα2/Kα1 = 0.5) operated at 30 kV and 10 mA. The diffracted intensity radiation measurements were made at scattering angles (2θ) between 5 °C and 80 °C with a step size of 0.01814 °/min. The Segal equation, Equation (3), was used to calculate the crystallinity index (χc) (Equation (3)):

(3) $$\chi c=\left(\frac{I_{002-}I_{am\;}}{I_{002}}\right)\times 100\%$$

where *I*_002_ is the peak intensity in the crystalline region (22°) and *I_am_* is the intensity in the amorphous region (17–19°).

### 2.11. Scanning Electron Microscopy (SEM) of the Fibers

The untreated fibers and those from the alkali- and acid-treated cellulosic pulps were analyzed with JEOL JSM IT-300 microscopy (JEOL Ltd., Tokyo, Japan). The samples were coated with Au in an ion beam coater for 10 s. Then, they were observed by SEM with an acceleration voltage of 20 kV. The thickness of the fibers was measured in triplicate and reported as the average ± standard deviation with the ImageJ software version 1.53a.

### 2.12. Statistical Analysis

The yield, holocellulose, extractive, and color analysis results for the alkali treatments were analyzed with a factorial design 2^2^, where the factors were the NaOH concentration and the processing time. The yield, holocellulose, extractive and color analysis results from the acid treatments were analyzed with a factorial design 2^3^, where the factors were the CH_3_COOH:H_2_O_2_ ratio, processing time, and temperature. All results are reported as the mean ± standard deviation. Statistical calculations were performed using the NCSS 2020 software program.

## 3. Results and Discussion

### 3.1. Proximate Chemical Composition of Mango’s Tegument

Mango’s tegument var. Tommy Atkins presented carbohydrate, ash, and fat content values of 95.58 ± 0.20%, 1.74 ± 0.04%, and 0.54 ± 0.06%, respectively. These values were similar to those reported by Torres-León et al. (2019) for mango var. Ataulfo (93.55%, 1.72%, 0.77%) [25]. Concerning the protein content, mango Tommy Atkins presented slightly lower values (2.13 ± 0.03%) than the reported for the Ataulfo variety (3.96%). Regarding crude fiber, mango Tommy Atkins presented a higher content than the reported for Ataulfo (73.80 ± 1.02% and 52.59%, respectively). The differences in chemical composition between the mango varieties can be attributed to the genetic varieties, growing conditions, and ripening stage [26].

### 3.2. Yield of Cellulosic Pulps

The yield of the cellulosic pulps is shown in Table 2. The lowest yield percentages were obtained with the alkaline treatments with 4% NaOH (1 and 2) compared to 2% NaOH. The increase in NaOH concentration decreased the fiber yield because the speed of the hydrolysis reaction increases proportionately with the hydroxide ion concentration [27], generating cellulosic pulp with less amorphous material. Regarding the acid treatment, the yields were higher than those obtained with alkali, except in treatments 8 and 12 (with a higher temperature and longer processing time). This behavior is attributed to the limited ability of acetic acid to break lignin, hemicellulose, and cellulose bonds, producing a cellulosic pulp with amorphous material. The delignification of samples with the highest concentrations of alkali and acid solutions was more efficient, yielding low values related to the degree of carbohydrate degradation (hemicelluloses) [18]. Fauzee and Othaman (2013) reported that the yield of the cellulosic pulp of a fruit treated with acid (CH_3_COOH) was slightly higher than that obtained with alkali (NaOH), at 34.8% and 31.0%, respectively, due to the hemicellulose content [28]. This behavior was also observed in our results. The application of physical and chemical treatments (or their combination) is required to separate the components of complex lignocellulosic materials, avoiding unwanted secondary products. Traditional procedures for obtaining cellulosic pulp from agro-industrial wastes are advantageous since the recovery and reuse of the produced liquors have been previously established. Therefore, it has a low environmental impact while attempting to reduce the impact of agro-industrial wastes in the vicinity of the mango fruit manufacturing site [29].

### 3.3. Effect of Alkali and Acid Treatments on the Color of the Cellulosic Pulps

**Figure 1:**
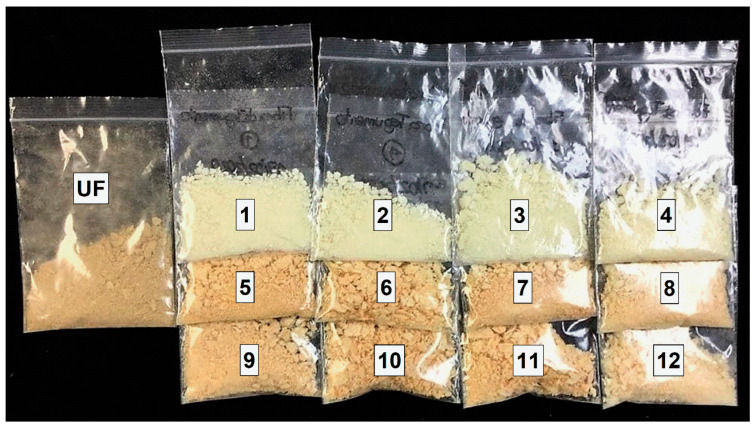
Appearance of cellulosic pulp obtained from alkali treatment (1–4), acid treatment (5–12), and untreated fibers (UF).

Images of the cellulosic pulp obtained from the alkali, acid, and untreated material are shown in Figure 1. The twelve treatments were evaluated to understand the modifications to the raw material that affected their appearance and to propose potential applications. According to Table 3, the alkali pulps presented a very pale-yellow color, close to white. The acidic pulps had a similar brown hue with an orange tone, like the untreated fibers. The untreated material had higher a* values (in the red direction) than those of the alkali-treated fibers, which is demonstrated by the significant differences (*p* < 0.05) and the highest values of ∆E (9–11). These effects can be easily appreciated by the naked eye as pale yellow with green shades. The qualitative attributes of color and hue presented differences between the untreated and the alkali-treated cellulosic pulps. With the H_2_O_2_ bleaching treatment, the lignin contained in the alkali cellulosic pulp was subjected to a bleaching process known as brightening [30]. However, the pulp was partially bleached since, in all the treatments, a large amount of lignin remained adhered, obtaining a yellow tone [31].

Regarding the comparison of the untreated fibers with the acid-treated fibers, in hue, all treatments were different from the untreated fiber (*p* < 0.05), except for treatment 12, with values between 76 and 83 related to an orange color. For ∆E, the value is between 1 and 5, and it is possible to confirm slight color changes compared to the untreated fibers. The acid cellulosic pulps had much more remaining lignin than the alkali treatments, and the bleaching did not contribute to obtaining a white color.

One of the potential applications of both cellulosic pulps is in the production of single-use items such as disposable plates, bowls, and cups. From the color point of view, the alkali cellulosic pulp could have consumer acceptance due to their clear pale-yellow relationship with clean surfaces. However, the acid cellulosic pulp could be associated with a less aggressive treatment that would allow greater acceptance because it resembles less refined pulp, related to a process with less environmental contamination.

### 3.4. Holocellulose, Lignin, and Extractives in the Untreated Fibers and Cellulosic Pulps

The alkali and acid treatments decreased the organic extractive values compared to the untreated fibers (*p* < 0.05), as shown in Table 4. The initial percentage of organic extractives in the mango tegument was high (4.64%). However, it was on the same order of magnitude as perennial crops (banana pseudostems 2.70%) and annual plants (amaranth 2.51%) [32]. The alkaline and acid treatments decreased the initial content of the organic extractives by more than 50%, showing the importance of pulping and bleaching in removing this type of substance.

Organic extractives are low-molecular-weight hydrophobic substances in wood and non-wood fiber pulps [33]. They can accumulate in the machinery and generate industrial problems such as low production levels, higher equipment costs, higher maintenance costs, and poor final product quality [34]. Removing extractives is essential for increasing inter-fiber bonding, which results in smoother and less porous paperboard [35].

The hot water extractives presented similar behavior to the organic extractives. They decreased (*p* < 0.05) in all treatments compared to the untreated fibers. Water extraction generates hydrolysates rich in sugars present in hemicellulose that were discarded. However, in acid treatments, processing time was an important factor. At 2 h, the water extractive content was higher than at 1 h because acetic acid causes the hydrolytic degradation of hemicelluloses. With prolonged contact time, cellulose is also degraded to a greater extent [36]. The hot water extractives in mango teguments were similar to those reported for cocoa pod husks (17.6%), another non-wood material. This content is higher than in wood fibers and directly affects the low yield of pulps [37]. According to Jiménez et al. (2007), reducing hot-water extractives allows for the obtainment of a good-quality raw material for developing paper sheets [38].

The holocellulose content is composed of cellulose and hemicelluloses. The holocellulose content increased in all treatments (*p* < 0.05). The alkali cellulosic pulp presented 73–75% of this compound without significant differences between treatments. Meanwhile, the acid cellulosic pulp presented a content of 78–88% with differences by time and processing temperature. The highest yield of holocellulose was obtained with treatments 5 and 9. The holocellulose content in cellulosic pulp is important since it is correlated with a higher pulp yield and better strength properties, which is convenient for the pulp and paper industry [39]. The developments of a bowl made from rice straw (holocellulose content 66.92%) and paper sheets made from cotton stalks (holocellulose content 72.86%) were technically feasible for both cases, with results similar to those made from traditional raw materials [35,38]. Regarding the lignin content, it did not present any changes for the studied treatments. The lignin content of the untreated fibers was 15.41 ± 2.61%, and it seems that the alkali treatments removed more hemicelluloses than lignin because the lignin content in the alkali pulp resulted in 21.96 ± 3.49% (treatment 3). In the case of the acid pulp, the lignin content was 17.71 ± 1.94 (treatment 9), similar to that of the untreated fibers, with a slight trend to increase, possibly because the hemicelluloses and lignin were removed at a lower degree than with the alkali treatment. This behavior agrees with the higher yield obtained for the acid pulp compared to that of the alkali pulp.

### 3.5. Fourier Transform Infrared Spectroscopy of the Cellulosic Pulps

**Figure 2:**
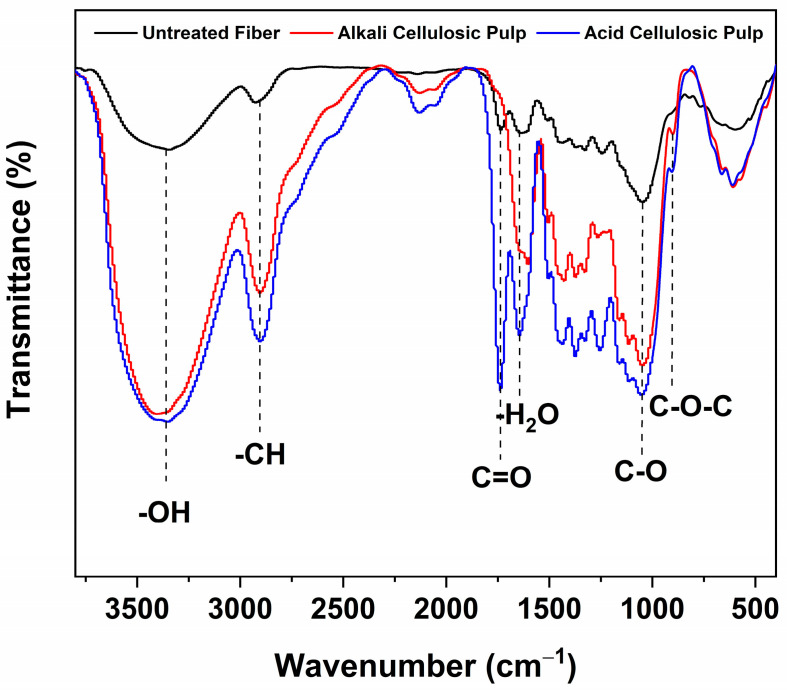
FTIR spectra of untreated fibers and cellulosic pulp (treatment 1 and treatment 5).

The influence of the hydrolysis of the bonds between cellulose, hemicellulose, and lignin in the cellulosic pulp is visible in the FTIR spectra shown in Figure 2. In all samples, the stretching vibration of hydroxyl groups (–OH) is observed at 3380 cm^−1^, associated with cellulose content. Our results agree with those reported by Ilangovan et al. (2020), where they worked with alkali-treated fibers of *Kigelia africana* fruits after bleaching, and the cellulose content was higher than that of the untreated fibers (71.6% and 55.15%, respectively) [24]. The C–H stretching vibration at 2900 cm^−1^ is present in all samples, with the lowest intensity in untreated fibers. It is a characteristic polysaccharide band, as is seen in natural fibers of hemp, sisal, jute, and kapok [40].

The band at 1734 cm^−1^ is related to carbonyl groups and it is attributed to the stretching of the acetyl ester and carbonyl aldehyde group (C=O) of hemicelluloses and lignin [41]. It is related to hemicellulose removal, which occurred to a lesser extent in the acid cellulosic pulp. According to Ohlmaier-Delgadillo et al. (2021), the pectin present in hemicelluloses had a high degree of esterification (52%) because the ratio between the esterified and non-esterified carboxyl groups was greater than 50% [42]. Chen et al. (2017) reported that hemicellulose was eliminated with 10% NaOH from the cellulosic pulp of bamboo [43]. Thus, we deduced that the alkali treatments reduced the hemicellulose content in the present work. The peaks between 1641 and 1649 cm^−1^ are related to the -O-H bending of the adsorbed water, characteristic of natural fibers [44]. The signal at 1048 cm^−1^ shows a higher intensity in acid and alkali pulp than in untreated fiber, which is related to the C-O groups of cellulose fibers [24]. Finally, the signal at 898 cm^−1^ indicates the presence of glycosidic bonds between monosaccharides from cellulose and hemicelluloses [40]. However, because of the molecular complexity and non-cellulosic components, this peak is less prominent in untreated fiber since the cellulose and hemicelluloses were not exposed. According to the FTIR spectra, the alkali treatment resulted in the most significant chemical modifications of the tegument fibers. Furthermore, the low yield in the alkali pulp can be attributed to the effectiveness of the treatment for successfully removing impurities (hemicelluloses).

### 3.6. Thermal Analysis of Cellulosic Pulps

**Figure 3:**
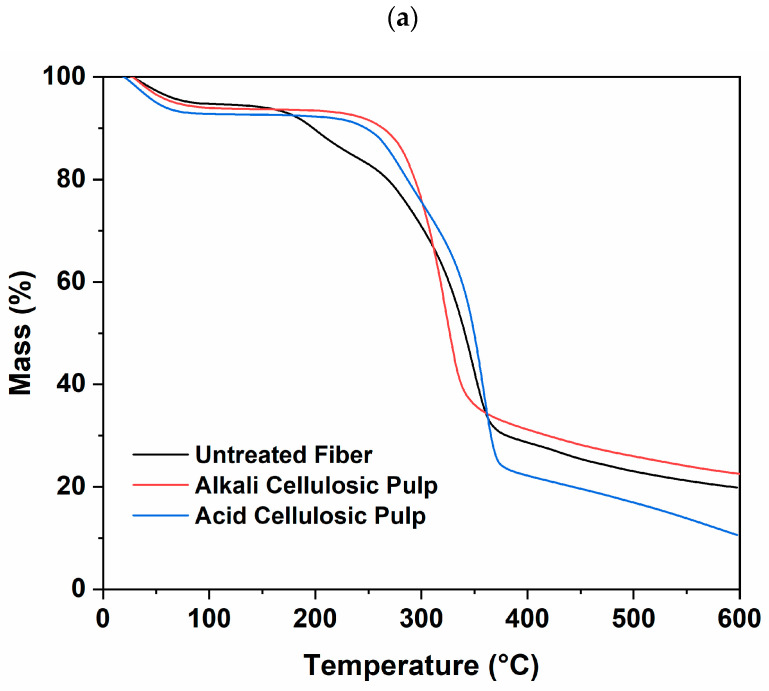

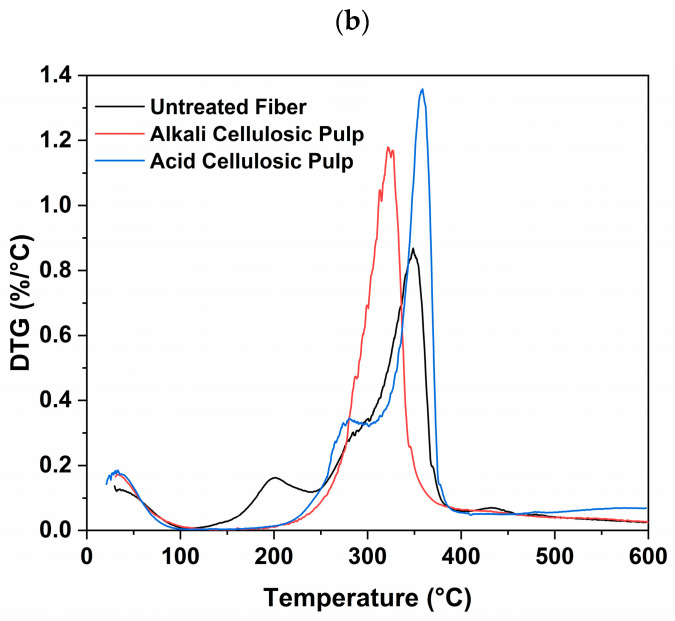
TGA (**a**) and DTG (**b**) of untreated fibers and cellulosic pulps. Cellulose *T_d_*: untreated fibers (347 °C), alkali (treatment 1) cellulosic pulp (316–321 °C), acid (treatment 5) cellulosic pulp (348–357 °C).

TGA and DTG thermograms for the untreated fiber and selected alkali and acid treatments are shown in Figure 3a,b. In general, the effect of the treatments showed the same behavior among the four alkali or the eight acid solutions. The remaining treatments are presented in Figure S2 (Supplementary Material). Untreated fibers and acid cellulosic pulp decomposed in three main stages. The first stage (<100 °C) is attributed to the loss of moisture from the fibers. The second stage (200 to 260 °C) relates to hemicellulose decomposition. The third stage (300 to 400 °C) presented the most extensive mass loss due to cellulose decomposition [45]. The maximum decomposition temperature of cellulose (Td) in the untreated fiber was 347 °C, while for the acid cellulosic pulps, it was 348–357 °C. In the case of alkali pulp, the treatments removed most of the hemicellulose contained in the fibers; hence, TGA showed two decomposition stages. The first is related to water evaporation (<100 °C), while the second is related to cellulose degradation. The Td of cellulose subjected to alkali treatment presented values between 316 and 321 °C. At the molecular level, both treatments decreased the hydrophobic character of the fibers due to the release of water molecules since the hydroxyl groups of the fibers were replaced by acetyl groups (acid treatment) and alkoxides (alkali treatment) [46].

The thermal stability may be reduced by alkali treatment due to the partial removal of hemicelluloses and lignin, decreased cellulose chain stuffing, cellulose depolymerization, the formation of short-length crystallites, and an increased surface area [15,47,48]. The acid-treated fibers remained the same as the untreated fibers, which may be explained by doping bulky groups onto the cellulose surface [48]. The thermal stability of the acid and alkali cellulosic pulps during the processing and usage temperatures of single-use disposable tableware (50–120 °C) demonstrates that plates, bowls, or cups made from these pulps may tolerate these processes where exposure to water could occur. Moreover, because of the thermal stability findings, acid and alkali cellulosic pulps could be used in the development of blends with biopolymeric materials, as they can withstand the processing temperatures of poly(lactic acid) and poly(hydroxyalkanoates) (150–200 °C) used in the extrusion and molding processes. This could result in potential uses as straws or rigid food packaging (trays) with reinforced structures.

### 3.7. X-ray Diffraction Analysis (XRD) of Cellulosic Pulps

**Figure 4:**
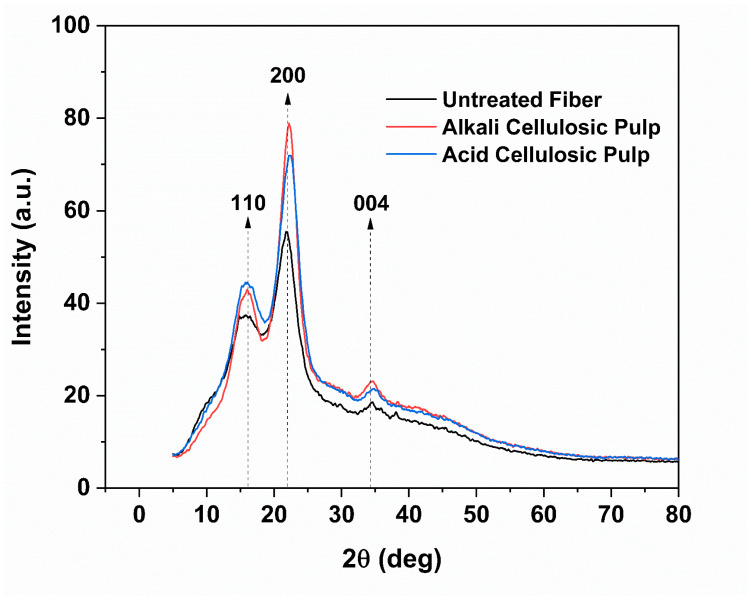
XRD spectra of untreated fibers and cellulosic pulps (treatment 1 and treatment 5).

Figure 4 shows the X-ray diffraction pattern of selected alkali and acid cellulosic pulps treatments and untreated fibers. All treatments had a similar effect on the crystallinity, as shown in Figure S3 (Supplementary Material). All samples presented the characteristic peak of cellulose I (22 ≤ 2θ ≤ 23) (plane 002), and the other peaks were between 13 and 18°, related to the 101 crystallographic plane of the cellulose and 34.5° (plane 004) [49,50]. Kathirselvam et al. (2019) reported that plants are rich in cellulose type Iβ and the XRD spectra presented four peaks at 15.3°, 16.2°, 22.8°, and 34.5°, similar to our results. Segal crystallinity (χc) was measured in the samples, where the untreated fibers showed a value of 46.5%, alkali cellulosic pulp values between 65.0% and 67.5%, and acid cellulosic pulp values between 55.4% and 62.9% [51]. Because of the removal of hemicellulose, the alkali treatment enhanced the crystallinity of the sample. In contrast, the acid treatment partially removed hemicellulose and left impurities on the surface. Therefore, both treatments increased the crystallinity of cellulose. The χc values presented in our research were similar to those reported by Bascón-Villegas et al. (2020) for lignocellulose nanofibers of tomato, eggplant, and pepper plants (56–60%), and the authors considered they were suitable for paperboard development [52]. High crystallinity provides higher strength, whereas low crystallinity enhances elongation, water intake, and chemical reaction sites [53].

### 3.8. Morphology of the Cellulosic Pulps

**Figure 5:**
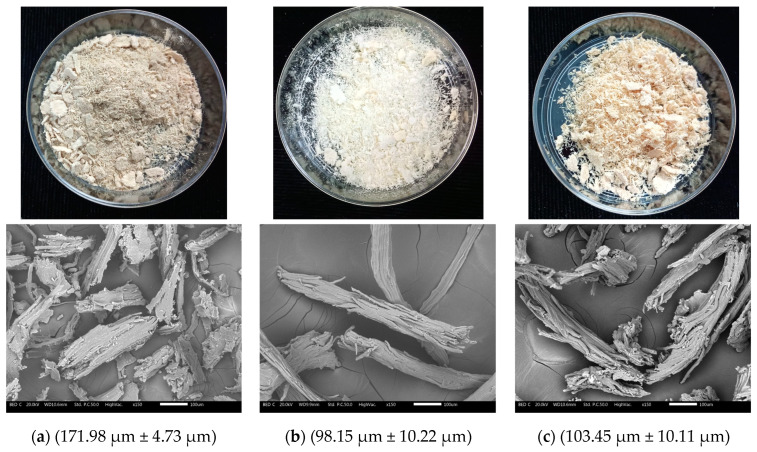
Image, SEM micrographs, and bundle diameter of untreated fibers (**a**), alkali-treated fibers (treatment 3) (**b**), and acid-treated fibers (treatment 9) (**c**).

Figure 5 shows images and SEM micrographs of the treated (treatments 3 and 9) and untreated fibers. Adherence to the bundle can be observed among the untreated fibers as in their natural state. Meanwhile, both treatments presented a degree of bundle breakage, showing a cleaner surface that allowed us to observe a decrease in the bundle diameter and individualization of the fibers being the most obvious after alkali treatment. Our results are consistent with those reported by Costa Santos et al. (2018), who evaluated piassava fibers treated with Ca(OH)_2_ and NaOH (2%) solutions, describing a better separation with the NaOH treatment compared to the untreated fibers [54]. The improvements in all treatments in the current work will result in a greater effective surface area of the fiber than the untreated fiber. They will contribute to creating fiber interactions in materials such as cardboard. Because of the hemicellulose remotion when using the NaOH reagent, fiber separation was greater in alkaline treatments than in acid treatments [55]. It is important to note that no fiber morphology differences were observed among the acid or alkaline treatments.

The alkali treatments generated a smooth texture because hemicelluloses and ashes were removed from the surface. A high NaOH concentration affected the fiber thickness since amorphous components such as hemicellulose were removed. Similar results were reported by Chen et al. (2017), who found that bamboo fibers treated with NaOH (25%) were thinner than fibers treated with NaOH (6%) [43]. Therefore, implementing treatments with higher NaOH concentration will improve the separation of tegument fibers by removing impurities like lignin and hemicellulose better. However, this will decrease the yield and fiber diameter.

In alkaline and acidic treatments, the fibers were separated from the bundles, suggesting that the treatments suit cellulosic pulp extraction. Our results are similar to those of Cordeiro et al. (2014), who reported that untreated fibers from mango’s tegument were packed due to the content of hemicelluloses and lignin that were partially removed with alkali treatment, promoting the breakdown of the fibers and pulp exposure [15]. In contrast, it is reported that alkali-treated cotton fibers presented a smoother surface than treated mango tegument fibers [56].

## 4. Conclusions

In the present investigation, a new source of cellulosic pulp was obtained from mango teguments through alkali and acid treatments. The alkali treatments produced cellulosic pulps with a light color, a lower yield, and a partial remotion of lignin and hemicellulose content due to the NaOH capacity to break ester linkages and the solubilization of these components. The acid treatments produced orange–brown cellulosic pulp with higher yields due to the lignin residues and a high holocellulose content. The acid cellulosic pulps were thermally more stable than the alkali pulps. Both treatments increased the crystallinity of the cellulosic pulp due to the removal of hemicellulose, with the alkali treatment having a more significant effect. The thermal stability of the acid and alkali cellulosic pulps at the application temperatures used in disposable tableware (50–120 °C) demonstrated that plates, bowls, trays, and cups could be produced, being dark-colored from the acid pulps and lighter-colored from the alkali pulps, reducing the demand for nonbiodegradable plastics when manufacturing these items. Another potential application is as a component of blends with biopolymers to make straws and rigid food packaging (trays) with reinforced structures. Finally, we suggest increasing the NaOH concentration to improve the lignin and hemicellulose remotion and obtain a cellulosic pulp with more potential for applications as cardboard articles.

## Figures and Tables

**Table 1:** Conditions of alkali and acid treatments.

Treatment	Number	Concentration Reagents	Processing Time (h)	Processing Temperature (°C)
Alkali NaOH (*w*/*v*)	1	4%	1	80
	2	4%	2	80
	3	2%	1	80
	4	2%	2	80
Acid Proportion CH_3_COOH:H_2_O_2_	5	1:1	1	60
	6	1:1	2	60
	7	1:1	1	70
	8	1:1	2	70
	9	2:1	1	60
	10	2:1	2	60
	11	2:1	1	70
	12	2:1	2	70

**Table 2:** Effect of alkali and acid treatments on the yield of cellulosic pulp.

Treatment	Number	Yield (% *w*/*w*)
Alkali NaOH (*w*/*v*)	1	37.64 ± 0.21 ^a^
	2	38.25 ± 1.83 ^a^
	3	42.24 ± 0.85 ^b^
	4	42.02 ± 0.45 ^b^
Acid Proportion CH_3_COOH:H_2_O_2_	5	46.97 ± 0.48 ^C^
	6	44.67 ± 1.44 ^B^
	7	45.12 ± 0.91 ^B^
	8	40.49 ± 0.31 ^A^
	9	47.98 ± 0.23 ^C^
	10	45.75 ± 0.32 ^B^
	11	45.95 ± 0.84 ^B^
	12	40.63 ± 0.83 ^A^

Different lowercase letters in the same column correspond to differences between alkali treatments (*p* < 0.05). Different uppercase letters in the same column correspond to differences between acid treatments (*p* < 0.05).

**Table 3:** Effect of alkali and acid treatments on color parameters for cellulosic pulp.

Treatment No.	Hue	∆E
Untreated fibers	82.61 ± 1.92 ^aC^	-
1	107.26 ± 0.49 ^d^	10.14 ± 0.13
2	104.88 ± 1.28 ^c^	9.76 ± 0.07
3	104.12 ± 1.78 ^c^	11.04 ± 0.19
4	101.58 ± 1.12 ^b^	9.80 ± 0.05
5	78.10 ± 1.42 ^B^	3.81 ± 1.27
6	79.68 ± 2.24 ^B^	3.48 ± 0.03
7	75.97 ± 1.37 ^A^	3.48 ± 0.02
8	84.96 ± 0.94 ^D^	3.04 ± 0.58
9	78.47 ± 0.61 ^B^	4.69 ± 0.11
10	77.78 ± 1.34 ^B^	3.16 ± 0.03
11	77.94 ± 0.17 ^B^	4.16 ± 0.32
12	83.24 ± 0.32 ^CD^	1.11 ± 0.57

Different lowercase letters in the same column correspond to differences between the alkali treatments and untreated fibers (*p* < 0.05). Different uppercase letters in the same column correspond to differences between the acid-treated and untreated fibers (*p* < 0.05).

**Table 4:** Effect of alkali and acid treatments on the chemical composition of cellulosic pulp.

Treatment No.	Organic Extractives (%)	Water Extractives (%)	Holocelullose Content (%) *
Untreated fibers	4.64 ± 1.02 ^bB^	17.39 ± 0.62 ^bC^	67.17 ± 1.63 ^a*aB*^
1	1.54 ± 0.02 ^a^	6.04 ± 0.26 ^a^	74.62 ± 0.74 ^b^
2	1.64 ± 0.17 ^a^	4.90 ± 0.46 ^a^	74.47 ± 0.74 ^b^
3	1.53 ± 0.09 ^a^	4.89 ± 0.42 ^a^	73.99 ± 0.86 ^b^
4	1.17 ± 0.09 ^a^	5.35 ± 1.10 ^a^	73.33 ± 1.55 ^b^
5	0.74 ± 0.047 ^A^	6.11 ± 0.64 ^A^	87.54 ± 0.82 *^cB^*
6	0.65 ± 0.07 ^A^	9.19 ± 1.23 ^B^	80.08 ± 0.37 *^bB^*
7	0.61 ± 0.04 ^A^	6.55 ± 0.42 ^A^	82.07 ± 0.68 *^cA^*
8	1.33 ± 0.28 ^A^	11.85 ± 0.30 ^B^	77.76 ± 0.71 *^bA^*
9	1.13 ± 0.12 ^A^	5.73 ± 0.49 ^A^	84.03 ± 0.60 *^cB^*
10	1.07 ± 0.35 ^A^	7.64 ± 0.01 ^B^	81.46 ± 0.18 *^bB^*
11	0.69 ± 0.07 ^A^	7.11 ± 0.38 ^A^	81.93 ± 0.03 *^cA^*
12	0.51 ± 0.04 ^A^	11.64 ± 0.27 ^B^	78.18 ± 0.72 *^bA^*

Different lowercase letters in the same column correspond to differences between the alkali treatments and the untreated fibers (*p* < 0.05). Different uppercase letters in the same column correspond to differences between the acid treatments and the untreated fibers (*p* < 0.05). * In holocellulose content, different lowercase, uppercase, and italics letters in the same column correspond to differences between the acid treatments and the untreated fibers (*p* < 0.05). Effect of time (*a*, *b*, or *c*). Effect of temperature (*A* or *B*).

## Data Availability

All data are included within this manuscript.

## Supplementary Materials

The following supporting information can be downloaded at https://www.mdpi.com/article/10.3390/polym15153163/s1: Figure S1: KBr tablets for FTIR analysis; Figure S2: TGA and DTG of untreated fibers, alkali (a,b), and acid (c,d) cellulosic pulps; Figure S3: XRD spectra of untreated fibers, alkali (a), and acid (b) cellulosic pulps.
