# Supplementary material for: Production and Characterization of Cellulosic Pulp from Mango Agro-Industrial Waste and Potential Applications
Source: Polymers (Basel). 2023 Jul 26;15(15):3163. doi: 10.3390/polym15153163 (PMC10421418; doi:10.3390/polym15153163)
Supplement: Supplementary file 1 [file polymers-15-03163-s001.zip › polymers-2489394-supplementary.pdf]

polymers

Article

Production and Characterization of Cellulosic Pulp from Mango Agro-Industrial Waste and Potential Applications

Maribel García-Mahecha [1], Herlinda Soto-Valdez [1,*], Elizabeth Peralta [1], Elizabeth Carvajal-Millan [2], Tomás Jesús Madera-Santana [1], María Guadalupe Lomelí-Ramírez [3] and Citlali Colín-Chávez [4]

[1] Coordinación de Tecnología de Alimentos de Origen Vegetal, Centro de Investigación en Alimentación y Desarrollo, A.C. (CIAD), Carretera Gustavo Enrique Astiazarán Rosas, No. 46, Col. La Victoria, Hermosillo 83304, Sonora, Mexico; maribel.garcia.dc19@estudiantes.ciad.mx (M.G.-M.); eperalta@ciad.mx (E.P.); madera@ciad.mx (T.J.M.-S.)
[2] Coordinación de Tecnología de Alimentos de Origen Animal, Centro de Investigación para Alimentación y Desarrollo, A.C. (CIAD), Carretera Gustavo Enrique Astiazarán Rosas, No. 46, Col. La Victoria, Hermosillo 83304, Sonora, Mexico; ecarvajal@ciad.mx
[3] Departamento de Madera, Celulosa y Papel del Centro Universitario de Ciencias Exactas e Ingenierías, Universidad de Guadalajara, Km 15.5 Carretera Guadalajara-Nogales, Zapopan 45220, Jalisco, Mexico; maria.lramirez@academicos.udg.mx
[4] Centro de Innovación y Desarrollo Agroalimentario de Michoacán, A.C. (CIDAM), Km 8 Antigua Carretera a Pátzcuaro s/n, Morelia 58341, Michoacán, Mexico; citllalina2982@gmail.com
* Correspondence: hsoto@ciad.mx; Tel.: +52-6622892400

Supplementary Materials

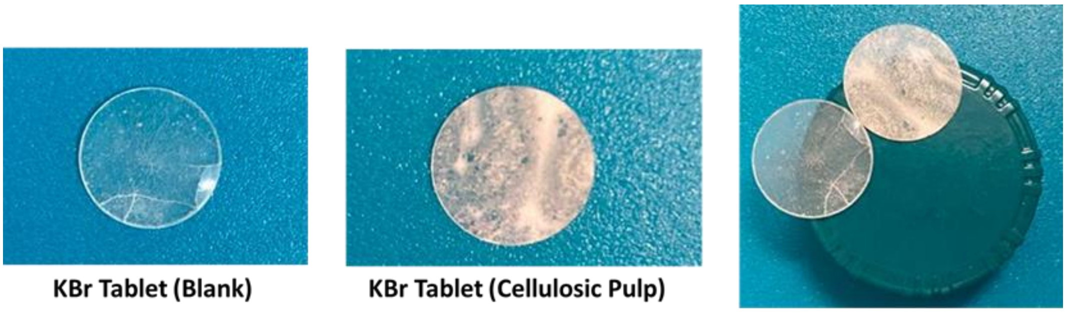

Figure S1. KBr tablets for FTIR analysis.

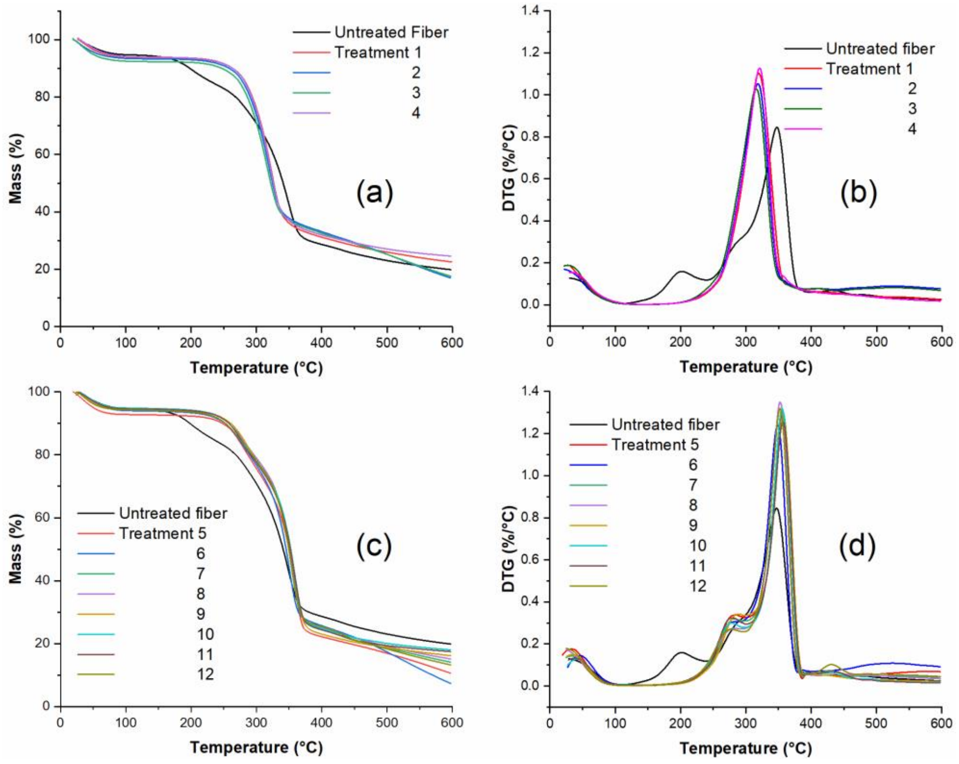

Figure S2. TGA and DTG of untreated fibers, alkali (a,b), and acid (c,d) cellulosic pulps.

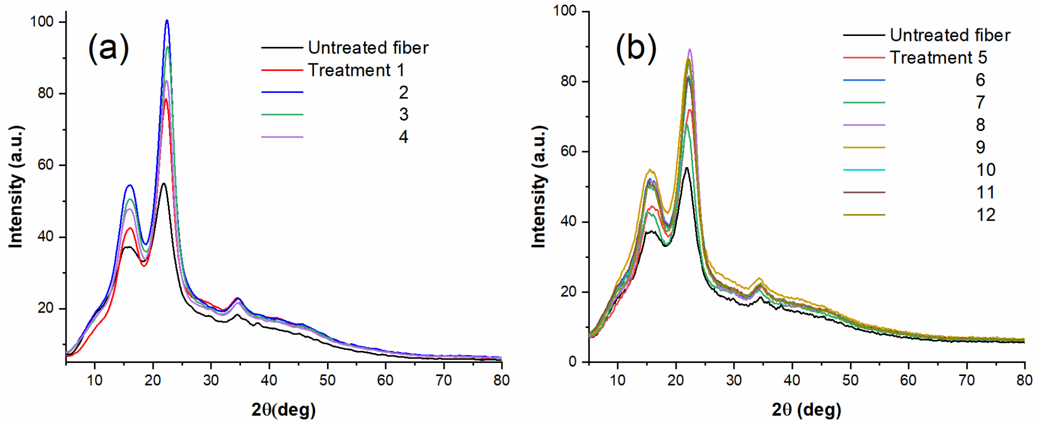

Figure S3. XRD spectra of untreated fibers, alkali (a), and acid (b) cellulosic pulps.
